# Impact of IL-6 and ferritin dynamics on clinical outcomes after anti-CD19 CAR-T cell therapy in diffuse large B-cell lymphoma patients

**DOI:** 10.3389/fimmu.2026.1906304

**Published:** 2026-08-19

**Authors:** Ling Gao, Yanyan Mao, Cai Sun, Yize Li, Xing Xing, Yuqing Miao, Chunling Wang, Hongfeng Ge, Ziyuan Shen, Wei Sang

**Affiliations:** 1 Department of Hematology, Jiangsu Province (Suqian) Hospital, Suqian, Jiangsu, China; 2 Department of Hematology, The Affiliated Hospital of Xuzhou Medical University, Xuzhou, Jiangsu, China; 3 Department of Hematology, Funing County People’s Hospital of Jiangsu Province, Yancheng, Jiangsu, China; 4 Department of Biostatistics, Johns Hopkins Bloomberg School of Public Health, Baltimore, MD, United States; 5 Department of Hematology, Yancheng First People’s Hospital, Yancheng, Jiangsu, China; 6 Department of Hematology, The First People’s Hospital of Huai’an, Huai’an, Jiangsu, China; 7 Department of Hematology, Bozhou People’s Hospital, Bozhou, Anhui, China; 8 Clinical Research Institute, The Affiliated Hospital of Xuzhou Medical University, Xuzhou, Jiangsu, China; 9 Cell Research and Translational Medicine Center, The Affiliated Hospital of Xuzhou Medical University, Xuzhou, Jiangsu, China; 10 Blood Diseases Institute, Xuzhou Medical University, Xuzhou, Jiangsu, China

**Keywords:** CAR-T, DLBCL, ferritin, interleukin-6, prognosis

## Abstract

**Background:**

Interleukin-6 (IL-6) and ferritin are routinely monitored during chimeric antigen receptor T-cell (CAR-T) therapy; however, their temporal dynamics and outcome-specific relevance remain inadequately characterized. This study aimed to evaluate the value of IL-6 and ferritin at multiple time points in patients with diffuse large B-cell lymphoma (DLBCL) receiving anti-CD19 CAR-T therapy.

**Methods:**

A total of 54 patients (median age 49.0 years; 64.8% male) were retrospectively analyzed. Post-infusion IL-6 and ferritin levels were measured on day 1, day 7, day 14, and the peak value was defined as the highest level within the first 14 days after infusion. Survival was evaluated using Cox proportional hazards regression, while logistic regression was used for binary outcomes, with Firth penalized regression performed as a sensitivity analysis. Spearman correlation was used to characterize relationships between IL-6, ferritin, and routine laboratory parameters.

**Results:**

CRS occurred in 68.5% of patients, and the objective response rate was 70.4%. IL-6 levels were significantly higher in patients with CRS at day 1 (*P* = 0.003) and at peak levels (*P* = 0.005). IL-6 on day 7 was associated with mortality (OR = 1.625, 95% CI: 1.076-2.737, *P* = 0.038). Ferritin was associated with CRS at all time points and showed an exploratory association with mortality, but not with treatment response. Day 7 IL-6 showed the numerically highest AUC for death status, whereas Day 14 ferritin showed the numerically highest AUC for CRS. IL-6 was negatively correlated with albumin at Day +1, Day +7, and peak, whereas ferritin was negatively correlated with RBC count at Day +1, Day +7, and peak.

**Conclusion:**

Serial IL-6 and ferritin measurements were associated with CRS-related inflammatory status. Day +7 IL-6 and Day +7 ferritin showed exploratory associations with mortality.

## 1 Introduction

Diffuse large B-cell lymphoma (DLBCL) is the most common and highly heterogeneous subtype of non-Hodgkin lymphoma (1). Chimeric antigen receptor T-cell (CAR-T) therapy has become a pivotal treatment for patients with relapsed or refractory (R/R) DLBCL, producing durable remissions in a subset of individuals (2). However, its clinical use is frequently accompanied by serious immune-related toxicities, most notably cytokine release syndrome (CRS) and immune effector cell-associated neurotoxicity syndrome (ICANS) (3). CRS arises from rapid and excessive immune activation, characterized by marked elevations in inflammatory cytokines such as interleukin-6 (IL-6), tumor necrosis factor-*α*, and interferon-*γ* (4, 5). These cytokines not only drive systemic inflammatory manifestations but also impair vascular endothelial integrity and disrupt the blood-brain barrier, potentially leading to severe organ dysfunction.

IL-6 plays a central role in the initiation and propagation of CAR-T-associated CRS (6, 7). It drives systemic inflammation by activating immune effector cells, enhancing vascular endothelial permeability, and inducing hepatic acute-phase protein synthesis, collectively contributing to high fever and hypotension. IL-6 has therefore become a key therapeutic target for CRS, and its receptor antagonist tocilizumab is widely used as first-line management (8). In addition to IL-6, ferritin, a marker of iron metabolism, has also been considered a potential indicator of CRS severity (9, 10). Elevated ferritin levels may reflect an immune-activated state resulting from macrophage activation syndrome (MAS) or systemic inflammation (11). Recent studies have demonstrated that increased IL-6 concentrations in DLBCL patients are significantly associated with inferior progression-free survival and overall survival (OS) (12, 13). Similarly, higher baseline ferritin levels have been reported to identify individuals at increased risk of severe toxicity and suboptimal clinical responses to CAR-T therapy (14). However, most existing studies have focused on single pre-treatment time points, with limited evaluation of the dynamic changes in IL-6 and ferritin levels at different time points post-CAR-T therapy and their associations with CRS occurrence and clinical outcomes.

This study aimed to characterize the dynamic changes of IL-6 and ferritin at predefined time points following CAR-T infusion and to evaluate their temporal relationships with CRS occurrence and subsequent clinical outcomes in patients with DLBCL.

## 2 Materials and methods

### 2.1 Study design

This retrospective study included DLBCL patients treated with anti-CD19 CAR-T therapy within the Huaihai Lymphoma Working Group (HHLWG) multicenter cohort between January 2019 and August 2024. All patients met the diagnostic criteria for DLBCL according to the World Health Organization classification (15) and underwent CAR-T therapy following standard lymphodepletion protocols. Eligible patients were required to be 18 years or older and have complete clinical records, including serial measurements of IL-6 and ferritin at Day 1, Day 7, and Day 14 post-infusion.

Patients were excluded if they: (1) lacked IL-6 or ferritin measurements at any of the prespecified time points, including Days +1, +7, and +14 after CAR T-cell infusion; (2) had active infection or uncontrolled autoimmune disease at baseline; (3) had concurrent hematologic malignancies or other active cancers; or (4) had end-stage hepatic or renal dysfunction.

This study was approved by the ethics committees of all participating centers within the HHLWG, and written informed consent was obtained from all participants.

### 2.2 Treatment protocol

All patients received anti-CD19 CAR-T cell therapy according to institutional protocols. The day of CAR-T-cell infusion was defined as Day 0. The lymphodepletion regimen consisted of fludarabine (30 mg/m²/day) and cyclophosphamide (300 mg/m^2^/day) administered on days -5 to -3 before CAR-T infusion. Patients were hospitalized for close monitoring of treatment-related adverse events, especially CRS. CRS was graded based on the American Society for Transplantation and Cellular Therapy (ASTCT) consensus criteria. In this study, corticosteroid and IL-6-targeted interventions were administered according to institutional protocols. For patients at high risk of ICANS, corticosteroids were administered early, typically initiated after CAR-T infusion upon the first occurrence of fever exceeding 38 °C (16).

### 2.3 Measurement of IL-6 and ferritin

Peripheral blood samples were collected at three predefined time points: Day +1, Day +7, and Day +14 after CAR-T-cell infusion, with the infusion day designated as Day 0. Serum levels of IL-6 and ferritin were measured using standardized clinical laboratory protocols. IL-6 concentrations were determined by chemiluminescence immunoassay, and ferritin levels were assessed by immunoturbidimetry. In addition to the scheduled Day +1, Day +7, and Day +14 measurements, peak IL-6 and ferritin values were defined as the highest available concentrations recorded within the first 14 days after CAR-T-cell infusion for each patient. These peak values were extracted from all clinically available laboratory records during this 14-day window and were not restricted to the three scheduled sampling time points.

### 2.4 Clinical outcomes

The primary clinical outcomes included CRS and OS. CRS was graded according to the ASTCT consensus criteria. CRS events occurring within 14 days after CAR-T cell infusion were recorded, and only the highest CRS grade per patient was used in the analysis. For the CRS severity-stratified analysis, patients were categorized into three groups according to the highest recorded CRS grade: no CRS, grade 1–2 CRS, and grade ≥3 CRS. Grade ≥3 CRS was defined as severe CRS according to the ASTCT consensus criteria. OS was defined as the time from CAR-T infusion to death from any cause or last follow-up. In addition, treatment response was assessed by PET-CT according to the Lugano 2014 criteria. Metabolic response was evaluated using the Deauville 5-point scale. Patients were categorized as having an objective response, including complete response (CR) and partial response (PR), or non-response (NR), including stable disease (SD) and progressive disease (PD).

### 2.5 Clinical and laboratory variables

Baseline clinical characteristics, including age, sex, Ann Arbor stage, Eastern Cooperative Oncology Group performance status (ECOG PS), and serum lactate dehydrogenase (LDH) levels, were collected from medical records.

Post-infusion laboratory parameters such as white blood cell count (WBC), hemoglobin (HGB), platelet count (PLT), and albumin (ALB) were also recorded.

### 2.6 Follow-up

Patients were followed from the date of CAR-T cell infusion until death or the last available follow-up. Survival status and disease progression were determined through review of inpatient and outpatient medical records, supplemented by telephone follow-up when necessary. All survival analyses were based on the originally defined database cutoff of April 30, 2025. Patients without a documented death on or before the cutoff date were censored at their last confirmed follow-up before the cutoff.

### 2.7 Statistical analysis

Normality of continuous variables was assessed using the Shapiro-Wilk test. Variables with non-normal distributions were summarized as medians with interquartile ranges (IQR). IL-6 and ferritin measurements were natural log-transformed for regression analyses to reduce right-skewness and the influence of extreme observations. Distributional changes before and after transformation were evaluated using Shapiro-Wilk tests, skewness statistics, and Q-Q plots. Descriptive statistics were presented as mean ± standard deviation or median with IQR for continuous variables, and frequencies with percentages for categorical variables. Between-group differences were evaluated using the Mann-Whitney U test for continuous variables, and chi-square test or Fisher’s exact test for categorical variables. For the CRS severity-stratified analysis, patients were classified into no CRS, grade 1–2 CRS, and grade ≥3 CRS groups. Because IL-6 and ferritin levels were markedly right-skewed, biomarker concentrations were summarized as medians with interquartile ranges and compared across the three CRS severity groups using the Kruskal-Wallis test. In addition, *P* values across the eight biomarker-timepoint comparisons were adjusted using the Benjamini-Hochberg false discovery rate method. Given that only six patients experienced grade ≥3 CRS, this severity-stratified analysis was considered exploratory, and no fully adjusted multivariable model was constructed for severe CRS. Overall survival was analyzed using Cox proportional hazards regression, with survival time calculated from the date of CAR-T-cell infusion to death from any cause or last follow-up. Hazard ratios (HRs), 95% confidence intervals (CIs), and *P* values were reported for IL-6 and ferritin levels at Day +1, Day +7, Day +14, and peak values. The proportional hazards assumption was assessed using Schoenfeld residual tests. Logistic regression models were used to evaluate associations between IL-6 or ferritin levels and binary outcomes, including CRS occurrence and treatment response. To evaluate potential multicollinearity between peak and fixed-timepoint biomarker measurements, variance inflation factors (VIFs) were calculated separately for the four IL-6 measurements and the four ferritin measurements after natural logarithmic transformation. A VIF of ≥10 was considered indicative of severe multicollinearity. Unadjusted models (Model 1) were first constructed, followed by adjusted models (Model 2) controlling for sex, age, Ann Arbor stage, ECOG PS, and LDH level. Given the limited number of outcome events relative to the number of covariates, multivariable Firth penalized logistic regression using the same adjustment variables was additionally performed as a sensitivity analysis to reduce small-sample bias and assess the stability of the conventional logistic regression estimates. Subgroup and interaction analyses were conducted to assess potential effect modification by clinical variables. The significance of interaction terms was evaluated using the likelihood ratio test. To account for multiple comparisons across biomarker-timepoint-outcome association analyses, Benjamini-Hochberg false discovery rate (FDR) correction was applied. Subgroup and sensitivity analyses were considered exploratory and hypothesis-generating.

Receiver operating characteristic (ROC) curve analysis was performed to evaluate the exploratory discriminative performance of IL-6 and ferritin measurements at Day +1, Day +7, Day +14, and peak values for mortality status at the database cutoff and any-grade CRS. The area under the ROC curve (AUC) and its 95% confidence interval were estimated using the DeLong method. The optimal cutoff value for each biomarker measurement was determined by maximizing the Youden index, and the corresponding sensitivity, specificity, positive predictive value, and negative predictive value were calculated. Pairwise comparisons of AUCs across the four measurement time points were performed using paired DeLong tests. To account for multiple pairwise comparisons, *P* values from the DeLong tests were adjusted using the Benjamini-Hochberg false discovery rate method. Cutoff values were reported on the original biomarker concentration scale, in pg/mL for IL-6 and ng/mL for ferritin.

Spearman correlation analysis was performed to examine the relationship between inflammatory markers (IL-6 and ferritin) and other laboratory indicators. Two-sided *P* values less than 0.05 were considered statistically significant. All statistical analyses were conducted using R software (version 4.5.1, https://www.r-project.org/).

## 3 Results

### 3.1 Baseline characteristics

A total of 54 patients were included, with a median age of 49.0 years (IQR, 38.0-54.8). Forty-seven patients (87.0%) were younger than 60 years, and 35 (64.8%) were male. Most patients had advanced Ann Arbor stage III/IV disease (42/54, 77.8%) (**Table 1**) and an ECOG performance status of ≥2 (39/54, 72.2%). Inflammatory biomarkers were monitored longitudinally. Median IL-6 levels were 14.0 pg/mL, 8.1 pg/mL, and 8.2 pg/mL on days 1, 7, and 14, respectively, and the median peak IL-6 level within the first 14 days was 22.4 pg/mL. Ferritin levels increased over time, with a median peak level of 716.5 ng/mL. CRS occurred in 37 patients (68.5%), including grade 1 CRS in 26 patients (48.1%), grade 2 CRS in 5 patients (9.3%), and grade ≥3 CRS in 6 patients (11.1%). ICANS occurred in 8 patients (14.8%), all of which were grade 1-2, and no grade 3-4 ICANS was observed. Thirty-eight patients achieved CR or PR, corresponding to an ORR of 70.4%, whereas 16 patients had no response. At a median follow-up of 25.7 months, 20 patients had died, and the Kaplan-Meier-estimated 5-year OS rate was 61.8%.

**Table 1:** Baseline characteristics in patients with DLBCL receiving CAR-T cell therapy.

Variables	Overall (n=54)
Age (median [IQR])	49.00 [38.00, 54.75]
Age (%)	
<60	47 (87.0)
≥60	7 (13.0)
Gender (%)	
male	35 (64.8)
female	19 (35.2)
Ann Arbor Stage (%)	
I/II	12 (22.2)
III/IV	42 (77.8)
ECOG PS (%)	
<2	15 (27.8)
≥2	39 (72.2)
LDH (median [IQR])	272.00 [195.50, 340.50]
IL6- day 1 (median [IQR])	14.00 [5.00, 21.82]
IL6-day 7 (median [IQR])	8.12 [4.40, 21.40]
IL6-day 14 (median [IQR])	8.19 [3.61, 24.25]
IL6-max (median [IQR])	22.40 [8.17, 39.95]
Fer- day 1 (median [IQR], ng/mL)	482.80 [265.83, 850.92]
Fer- day 7 (median [IQR], ng/mL)	552.25 [255.20, 1112.50]
Fer- day 14 (median [IQR], ng/mL)	610.15 [254.52, 1331.50]
Fer-max (median [IQR], ng/mL)	716.50 [366.20, 1452.50]
CRS grade (%)	
No CRS	17 (31.5)
Grade 1	26 (48.1)
Grade 2	5 (9.3)
Grade ≥3	6 (11.1)
Treatment response (%)	
NR	16 (29.6)
CR/PR	38 (70.4)
Status	
Alive	34 (63.0)
Dead	20 (37.0)

IQR, interquartile range; LDH, lactate dehydrogenase; IL6, interleukin-6; Fer, ferritin; CRS, cytokine release syndrome; ECOG-PS, Eastern Cooperative Oncology Group performance status; NR, non-response; CR/PR, complete response/partial response.

### 3.2 Between-group differences in IL-6 and ferritin

**Figure 1:**
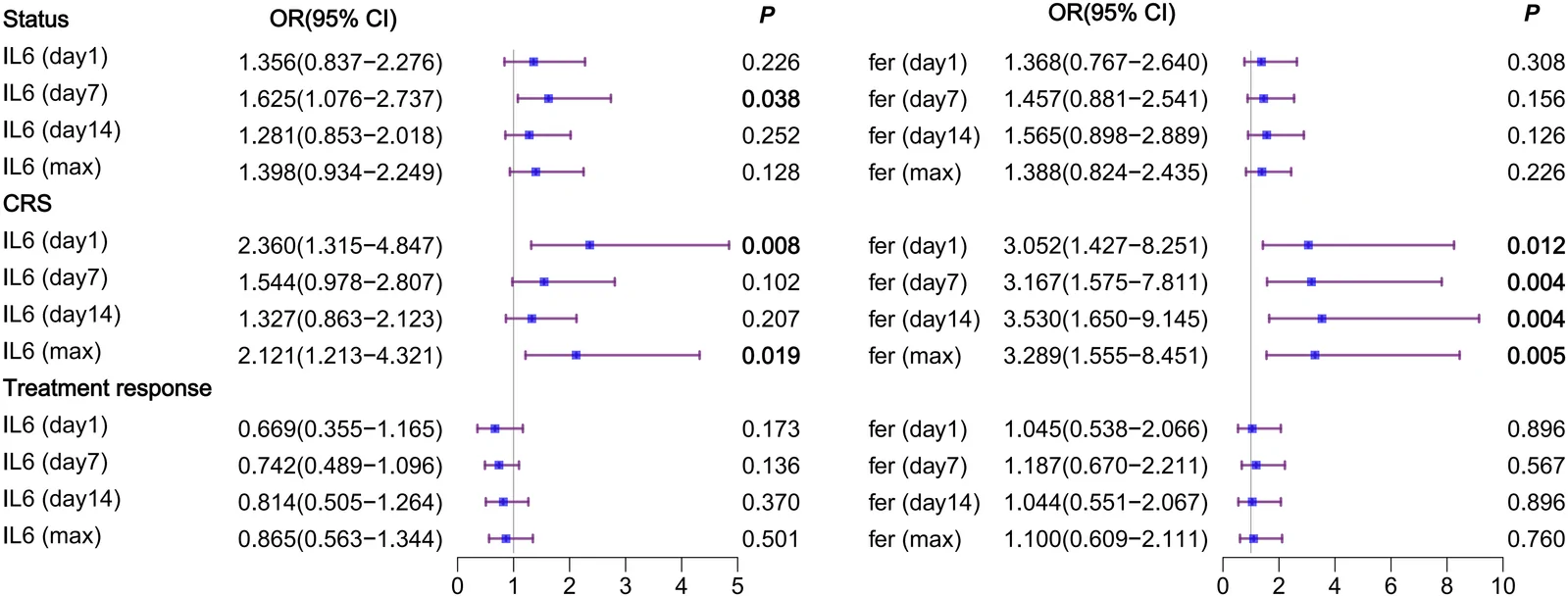
Comparisons of IL-6 levels across clinical subgroups at different time points. Natural log-transformed IL-6 levels were compared according to survival status, CRS occurrence, and treatment response at Day 1, Day 7, Day 14, and peak level within the first 14 days. The original IL-6 concentration was measured in pg/mL.

IL-6 levels were compared between clinical subgroups at days 1, 7, and 14, as well as at peak levels. Patients who experienced CRS exhibited higher IL-6 levels on day 1 (*P* = 0.003, **Figure 1**) and at peak (*P* = 0.005) compared to those without CRS. Additionally, IL-6 levels on day 7 were elevated in patients who died compared to survivors (*P* = 0.012). No significant differences in IL-6 levels were observed between responders (CR/PR) and non-responders at any time point (all *P* > 0.1), nor were there differences at other time points by survival status or CRS status.

**Figure 2:**
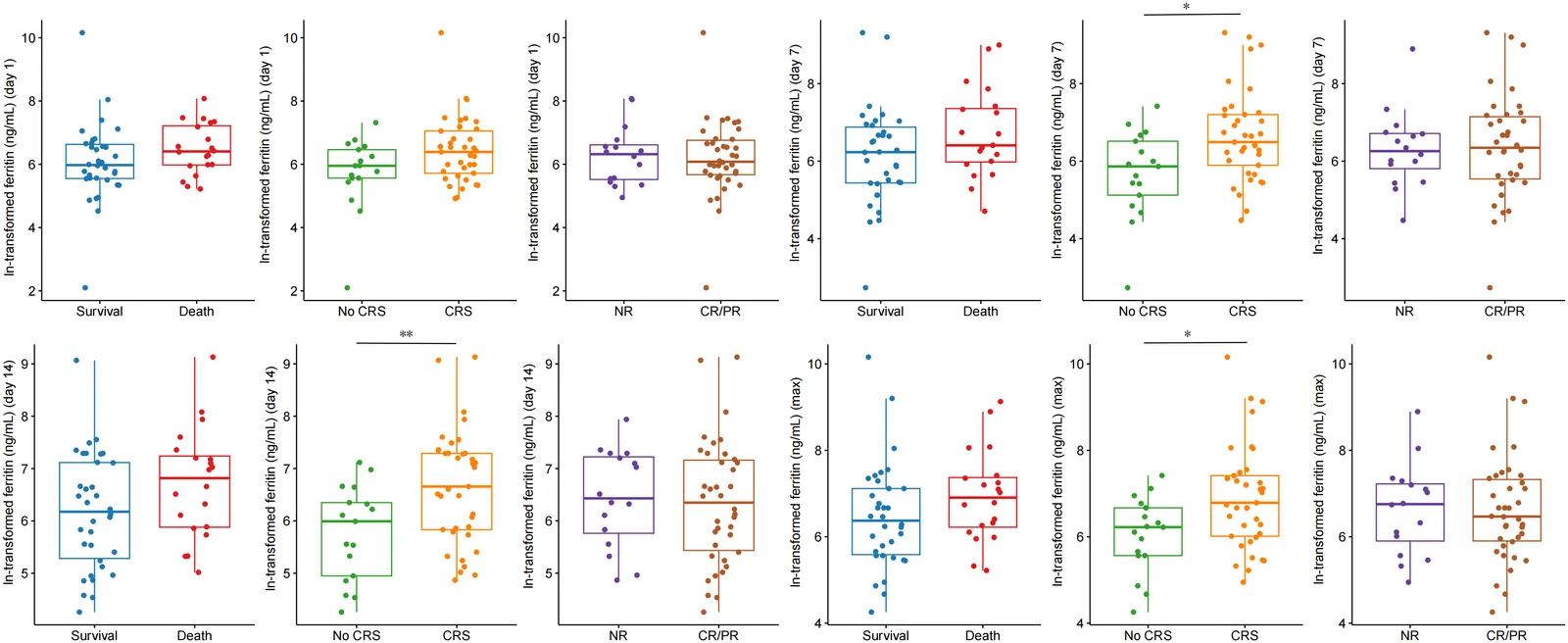
Comparisons of ferritin levels across clinical subgroups at different time points. Natural log-transformed ferritin levels were compared according to survival status, CRS occurrence, and treatment response at Day 1, Day 7, Day 14, and peak level within the first 14 days. The original ferritin concentration was measured in ng/mL. *P < 0.05; **P < 0.01.

Ferritin levels showed a significant association with CRS occurrence. Patients with CRS had higher ferritin levels at day 7 (*P* = 0.012, **Figure 2**), day 14 (*P* = 0.005), and peak levels (*P* = 0.020) compared to those without CRS. However, no significant differences in ferritin levels were observed between survival groups (all *P* > 0.1) or between response groups (CR/PR vs. NR, all *P* > 0.5) at any of the assessed time points.

In an exploratory analysis stratified by CRS severity, patients were categorized as having no CRS (n = 17), grade 1–2 CRS (n = 31), or grade ≥3 CRS (n = 6). IL-6 levels differed significantly across the three groups on day 1 (*P* = 0.007, FDR-adjusted *P* = 0.027), day 7 (*P* = 0.037, FDR-adjusted *P* = 0.042), and at peak (*P* < 0.001, FDR-adjusted *P* = 0.002), whereas no significant difference was observed on day 14 (*P* = 0.386). Ferritin levels differed significantly across CRS severity groups on day 1 (*P* = 0.034, FDR-adjusted *P* = 0.042), day 7 (*P* = 0.030, FDR-adjusted *P* = 0.042), day 14 (*P* = 0.010, FDR-adjusted *P* = 0.028), and at peak (*P* = 0.017, FDR-adjusted *P* = 0.035) (**Supplementary Table 1**).

### 3.3 Associations between IL-6, ferritin and outcomes

In univariable Cox proportional hazards analyses, higher IL-6 levels on Day 7 were associated with an increased hazard of all-cause mortality (HR = 1.368, 95% CI: 1.042-1.795, *P* = 0.024). Ferritin levels on Day 7 were also associated with an increased mortality hazard (HR = 2.057, 95% CI: 1.064-3.974, *P* = 0.032, **Supplementary Table 3**). No significant survival associations were observed for IL-6 or ferritin at the other fixed time points.

**Figure 3:**
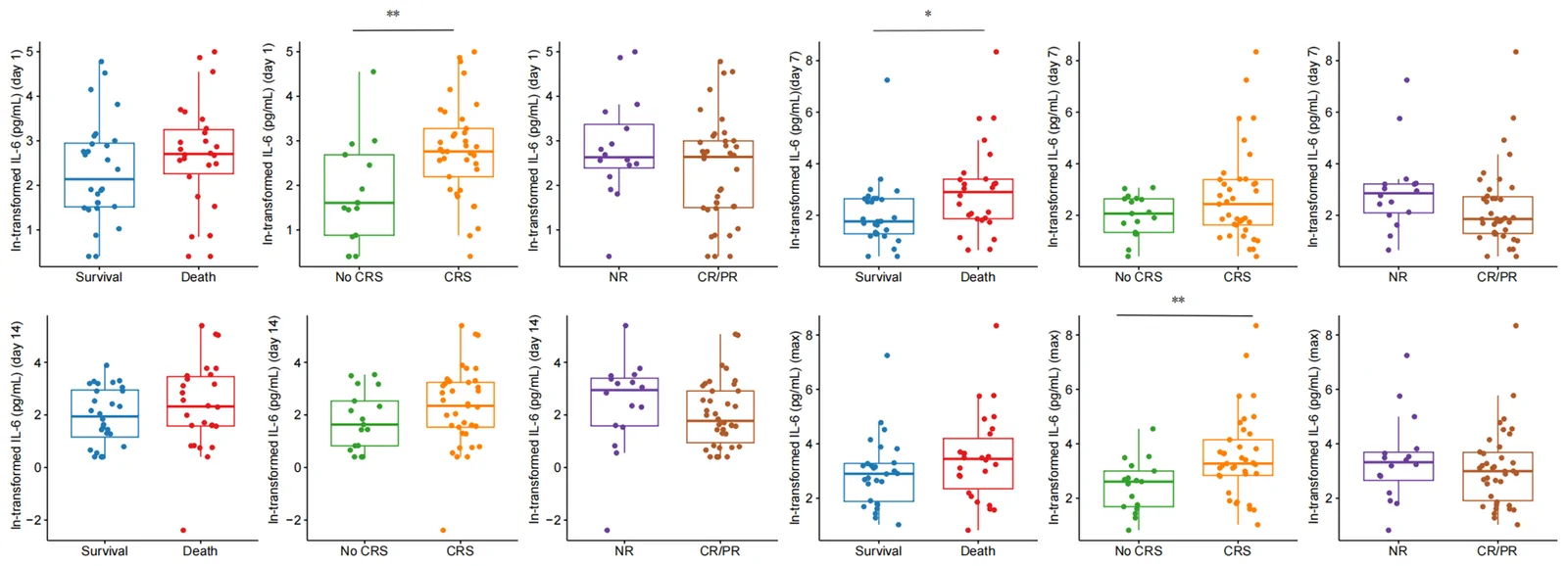
Forest plots displaying adjusted odds ratios and 95% confidence intervals for natural logtransformed IL-6 levels in relation to clinical outcomes. ORs represent the change in odds per 1-unit increase in the natural logtransformed IL-6 level, corresponding to an approximately 2.718-fold increase in the original IL-6 concentration. All models were adjusted for age, sex, Ann Arbor stage, ECOG performance status, and LDH level. *P < 0.05; **P < 0.01.

In the conventional multivariable logistic analysis based on vital status at the last follow-up, higher Day 7 IL-6 was associated with mortality (OR = 1.625, 95% CI: 1.076–2.737, *P* = 0.038; **Figure 3**). However, in the multivariable Firth penalized sensitivity analysis, the effect direction remained positive, but the association was no longer statistically significant (OR = 1.375, 95% CI: 0.908-2.244, *P* = 0.136). The associations of Day 1 and peak IL-6 with CRS remained statistically significant in the Firth models. Ferritin levels at Day 1, Day 7, Day 14, and peak also remained significantly associated with CRS. No significant associations with treatment response were observed in the Firth analyses. Complete results are presented in **Supplementary Table 5**.

In the CRS group, IL-6 levels were elevated on day 1 and at peak. No significant associations were observed between IL-6 and treatment response. Ferritin levels showed consistent associations with CRS at all time points: day 1 (OR = 3.052, 95% CI: 1.427-8.251, *P* = 0.012), day 7 (OR = 3.167, 95% CI: 1.575-7.811, *P* = 0.004), day 14 (OR = 3.530, 95% CI: 1.650-9.145, *P* = 0.004), and peak (OR = 3.289, 95% CI: 1.555-8.451, *P* = 0.005). In the adjusted logistic regression analyses based on vital status at the database cutoff, ferritin levels at all assessed time points were not significantly associated with mortality status. No significant associations between ferritin and treatment response were observed. In the auxiliary collinearity analysis, VIF values ranged from 1.349 to 2.129 for IL-6 measurements and from 3.781 to 8.736 for ferritin measurements.

### 3.4 Predictive ability of IL-6 and ferritin for mortality and CRS

**Figure 4:**
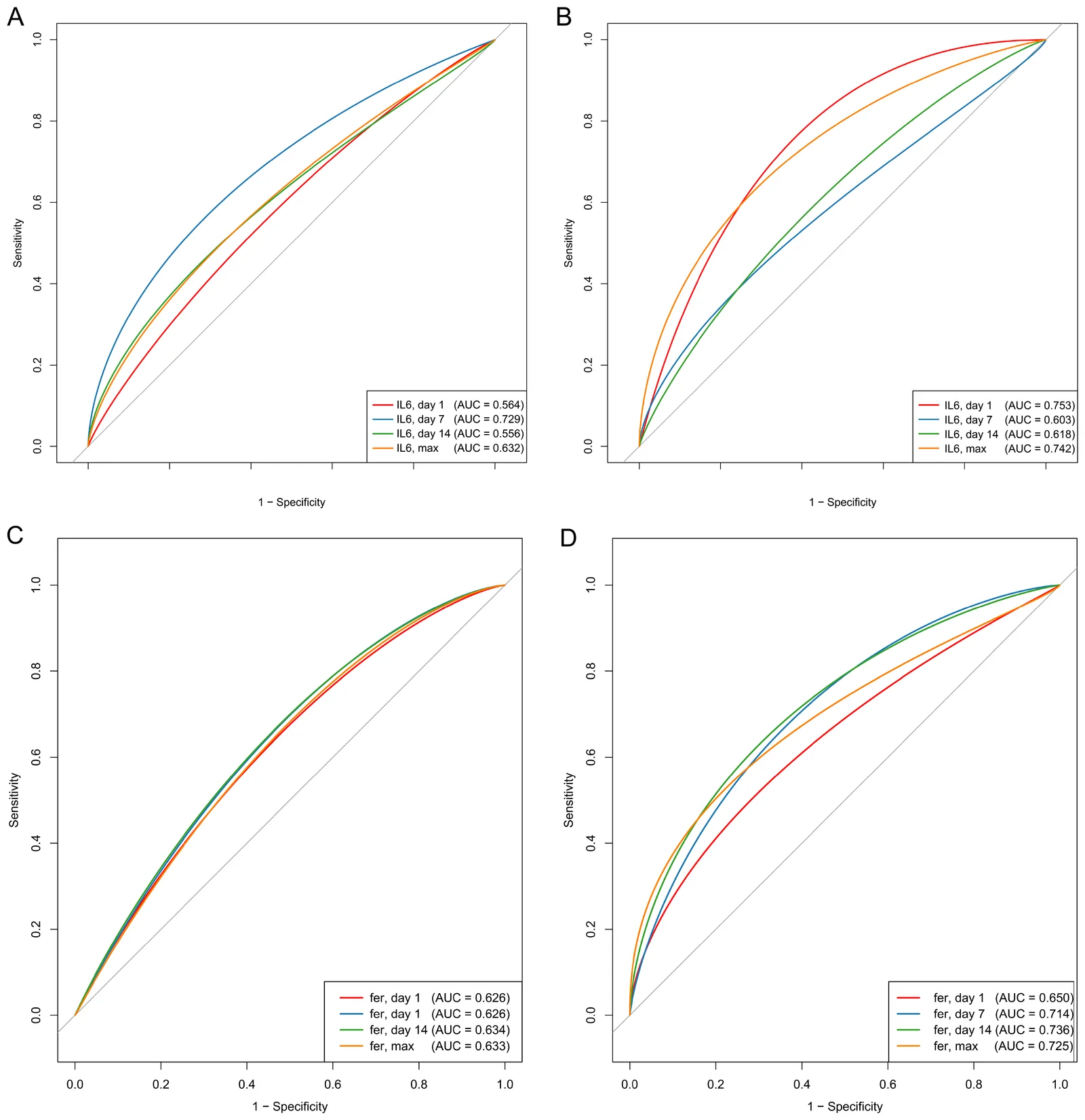
Receiver operating characteristic (ROC) curves showing the predictive performance of IL-6 and ferritin levels at Day 1, Day 7, Day 14, and peak values for mortality and CRS. **(A)** IL-6 for mortality. **(B)** IL-6 for CRS. **(C)** Ferritin for mortality. **(D)** Ferritin for CRS.

ROC curve analyses were performed to evaluate the exploratory discriminative performance of serial IL-6 and ferritin measurements for death status at the last follow-up and any-grade CRS. Day 7 IL-6 showed the numerically highest AUC for death status (AUC = 0.729, 95% CI: 0.589-0.868, **Figure 4**). Its AUC was nominally higher than those of Day 1 IL-6 (DeLong *P* = 0.033) and Day 14 IL-6 (DeLong *P* = 0.037), although these differences did not remain statistically significant after FDR correction (both FDR-adjusted *P* = 0.112).

For any-grade CRS, Day 1 and peak IL-6 showed the numerically highest AUCs of 0.753 (95% CI: 0.606-0.900) and 0.742 (95% CI: 0.601-0.882), respectively. Ferritin on Day 14 showed the numerically highest AUC for CRS (AUC = 0.736, 95% CI: 0.601-0.871). However, no pairwise AUC comparison for CRS remained statistically significant after FDR correction. Complete ROC performance metrics, optimal cutoffs, and pairwise DeLong comparisons are provided in **Supplementary Table 4**.

### 3.5 Correlation between IL-6, ferritin and clinical parameters

**Figure 5:**
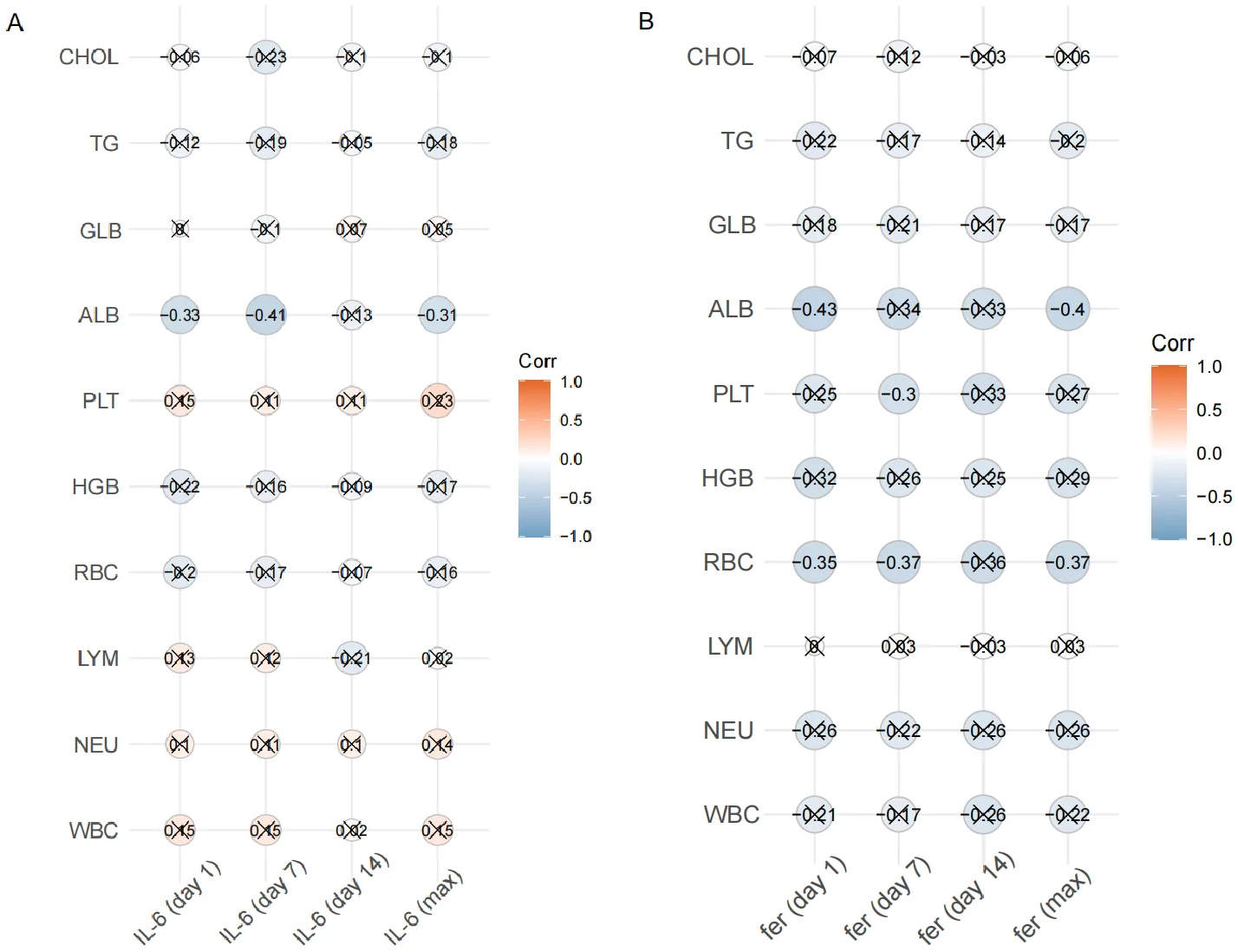
Correlation heatmaps of IL-6 and ferritin with clinical laboratory parameters at different time points after CAR-T cell infusion. **(A)** Spearman correlation between IL-6 levels (day 1, day 7, day 14, and maximum) and routine clinical laboratory indicators. **(B)** Spearman correlation between ferritin levels (day 1, day 7, day 14, and maximum) and the same set of indicators. Crossed cells indicate correlations that were not statistically significant. CHOL, cholesterol; TG, triglycerides; GLB, globulin; ALB, albumin; PLT, platelet count; HGB, hemoglobin; RBC, red blood cell count; LYM, lymphocyte count; NEU, neutrophil count; WBC, white blood cell count; IL-6, interleukin-6; Fer, ferritin.

IL-6 levels were significantly negatively correlated with ALB on Day +1, Day +7, and at peak, with the strongest correlation observed on Day +7 (**Figure 5**). Ferritin levels were significantly negatively correlated with RBC count on Day +1, Day +7, and at peak; with ALB on Day +1 and at peak; and with PLT count on Day +7.

## 4 Discussion

In this study, serial IL-6 and ferritin measurements showed distinct associations with CAR T-cell-related toxicity and clinical outcomes. Given the relatively low incidence and mild severity of ICANS in our cohort, we focused primarily on the potential associations of IL-6 and ferritin with CRS and other major safety outcomes. Ferritin levels were consistently associated with CRS across the assessed time points. In univariable time-to-event analyses, Day 7 IL-6 and Day 7 ferritin showed nominal associations with all-cause mortality, while the association between peak ferritin and mortality varied over time.

Higher Day +7 IL-6 showed an association with mortality in the conventional logistic model; however, this association was attenuated and no longer statistically significant in the Firth sensitivity analysis. This time point is beyond the typical early IL-6 peak that accompanies acute CRS, suggesting that persistent elevation may indicate prolonged immune activation or impaired cytokine clearance, particularly in patients with hepatic or systemic dysfunction (17, 18). Given the short biological half-life of IL-6, continued elevation at day 7 may reflect an unresolved or secondary inflammatory phase involving monocyte-macrophage activation, endothelial injury, or cytokine-mediated organ damage, as observed in hyperinflammatory conditions such as hemophagocytic lymphohistiocytosis (HLH) and severe CRS. While previous studies have mainly focused on IL-6 elevations within the first 72 hours as predictors of severe CRS and neurotoxicity, few have evaluated the prognostic significance of IL-6 beyond this acute window (11). Our findings suggest that day 7 represents a clinically meaningful inflection point at which IL-6 may no longer solely reflect acute cytokine toxicity but instead signal ongoing inflammation or tissue injury that predisposes patients to late complications and mortality. The predictive performance at this time point further supports the potential utility of day-7 IL-6 as an early biomarker of mortality risk.

Ferritin levels in our cohort displayed a markedly right-skewed distribution, with a subset of patients exhibiting disproportionately elevated concentrations. This pattern likely reflects a biologically heterogeneous inflammatory response, wherein a minority of individuals develop pronounced macrophage activation and iron dysregulation (19). Consistently, ferritin was significantly associated with CRS at all measured time points, suggesting that elevated ferritin reflects not only an acute-phase reaction but also a sustained inflammatory state closely linked to immune-mediated toxicity (20). This finding aligns with prior reports identifying hyperferritinemia as a hallmark of macrophage activation syndromes and severe cytokine-driven conditions such as HLH (21–23). Ferritin showed no significant association with treatment response. Day +7 ferritin showed a nominal univariable association with mortality and peak ferritin demonstrated a time-varying mortality association. A plausible explanation for its prolonged elevation is ferritin’s relatively long biological half-life and slower clearance kinetics, particularly in patients with hepatic dysfunction or ongoing inflammatory burden (24). Ferritin synthesis is also induced by pro-inflammatory cytokines such as IL-1β and TNF-α, which may remain elevated even after IL-6 levels begin to decline. These factors likely contribute to the broad temporal window over which ferritin remains elevated in patients with CRS. While elevated ferritin has been described in severe CRS, few studies have systematically characterized its dynamic behavior across multiple time points. Our findings highlight ferritin as a valuable complementary biomarker for real-time monitoring of immune toxicity during CAR-T therapy.

The correlations between IL-6, ferritin, and routine laboratory parameters may provide exploratory clues regarding CAR-T–related immune dysregulation; however, these findings should be interpreted cautiously because correlation analyses cannot establish causal or mechanistic relationships. IL-6 showed negative correlations with albumin on Day +1, Day +7, and at peak, with the strongest correlation observed on Day +7, a pattern compatible with its known role in modulating the hepatic acute-phase response and suppressing albumin synthesis during inflammation (25, 26). Ferritin showed negative correlations with RBC count on Day +1, Day +7, and at peak, which may reflect systemic inflammation, impaired erythropoiesis, hemophagocytic activity, or disease-related hematologic vulnerability, although these potential explanations remain speculative (27). Ferritin also showed inverse correlations with albumin at selected time points and with platelet count on Day +7. These associations may indicate a systemic inflammatory phenotype and could be interpreted in the context of marrow suppression, consumptive processes, or endothelial injury observed in severe CRS and HLH-related hyperinflammatory conditions (28–30).

Serial IL-6 and ferritin monitoring may provide supportive information for risk-adapted management during the early post-CAR-T period. The moderate discriminative performance observed in this study suggests that these biomarkers should not be used as standalone decision-making tools or as substitutes for established CRS grading and management algorithms. However, persistently elevated IL-6 on Day +7 or rising ferritin on Day +14 may help identify patients who require closer clinical surveillance, including more frequent assessment of fever, hemodynamic instability, oxygen requirement, hepatic function, coagulation status, cytopenias, and infection-related complications. In practice, these biomarker trends may serve as adjunctive signals to prompt earlier multidisciplinary evaluation and intensified organ monitoring in high-risk patients. Decisions regarding tocilizumab, corticosteroids, or other anti-inflammatory interventions should continue to be based on CRS grade, organ dysfunction, and institutional treatment protocols, with IL-6 and ferritin dynamics used as complementary rather than definitive criteria.

In this study, we systematically evaluated the dynamic changes of IL-6 and ferritin across multiple predefined time points and peak values, and analyzed their associations with both CRS and mortality. Several limitations should be acknowledged. First, the retrospective multicenter design and limited sample size may have introduced selection bias and reduced the precision and generalizability of the effect estimates. Although patients with major baseline inflammatory conditions were excluded to reduce confounding from non-CAR-T-related inflammation, this may have narrowed the inflammatory spectrum of the cohort and attenuated the observed associations between IL-6 or ferritin and clinical outcomes. The direction and magnitude of this potential bias could not be quantified. Second, several relevant CAR-T-specific variables, including prior lines of therapy, bridging treatment, CAR-T product subtype, tumor burden, and toxicity-directed interventions, were not consistently recorded and therefore could not be fully adjusted for, leaving the possibility of residual confounding. Third, pre-infusion IL-6 and ferritin measurements were not consistently available at standardized time points across participating centers, precluding a reliable assessment of their associations with subsequent CRS severity. In addition, peak biomarker values were derived from clinically available measurements obtained at nonstandardized testing frequencies, and the limited number of grade ≥3 CRS events restricted severity-specific analyses. Finally, only IL-6 and ferritin were evaluated, and the findings were not supported by mechanistic investigations or external validation.

In summary, serial IL-6 and ferritin measurements were associated with CRS-related inflammatory status in patients with DLBCL receiving CAR-T-cell therapy. Day +7 IL-6 and Day +7 ferritin showed exploratory associations with mortality in univariable or conventional regression analyses, but the robustness of these findings was limited by the small sample size, limited event volume, and sensitivity to model specification. These biomarkers should therefore be regarded as adjunctive monitoring indicators rather than standalone prediction or treatment-decision tools. Prospective, large-scale multicenter studies with standardized sampling and external validation are needed to confirm their clinical utility.

## Data Availability

Publicly available datasets were analyzed in this study. This data can be found here: The datasets generated and/or analyzed during the current study are available from the corresponding author on reasonable request.
