# Supplementary material for: Impact of IL-6 and ferritin dynamics on clinical outcomes after anti-CD19 CAR-T cell therapy in diffuse large B-cell lymphoma patients
Source: Front Immunol. 2026 Aug 19;17:1906304. doi: 10.3389/fimmu.2026.1906304 (PMC13539416; doi:10.3389/fimmu.2026.1906304)
Supplement: Supplementary file 1 [file DataSheet1.pdf]

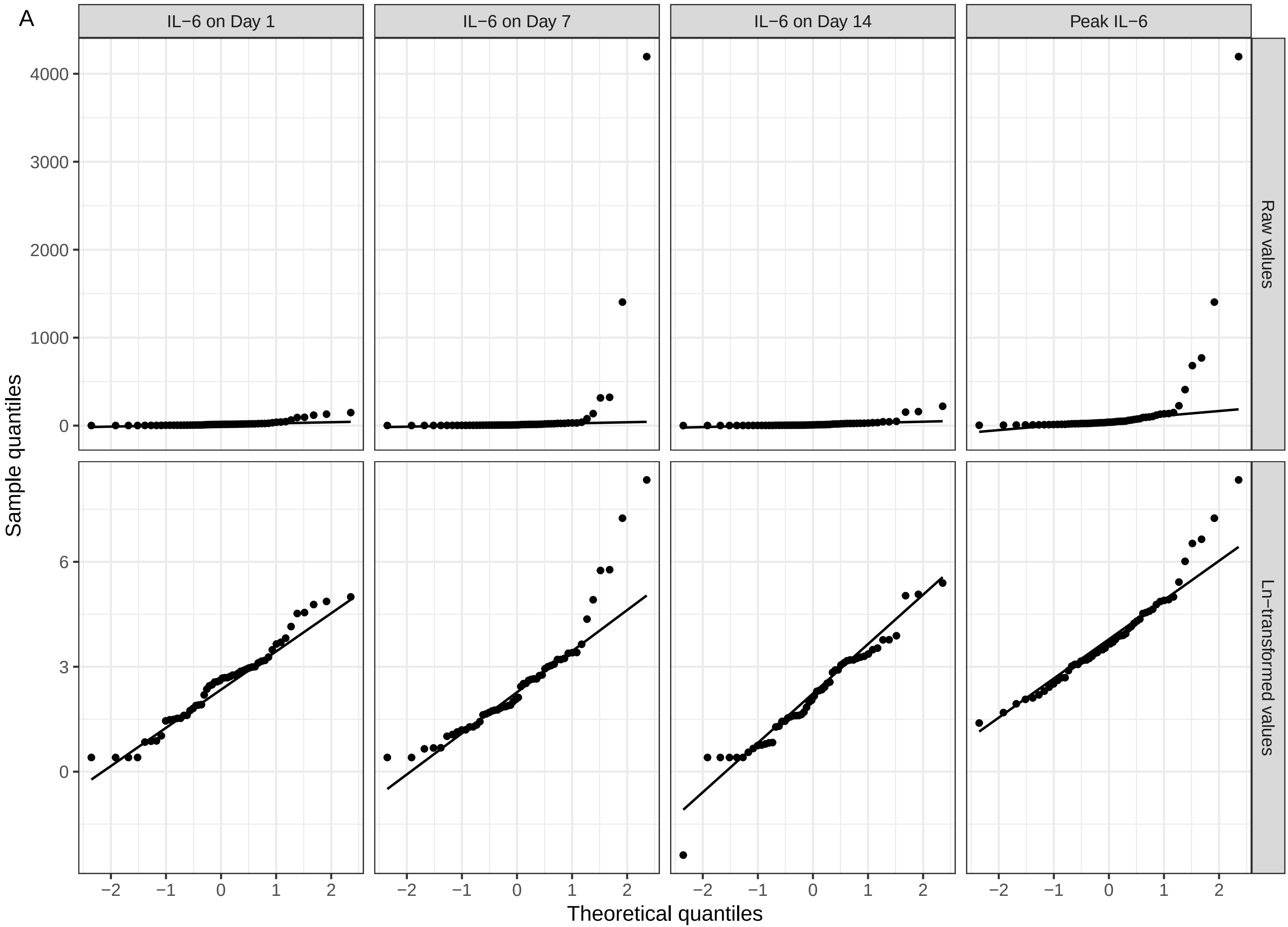

A
IL−6 on Day 1
IL−6 on Day 7
IL−6 on Day 14
Peak IL−6
Raw values
Ln−transformed values
Sample quantiles
Theoretical quantiles
4000
3000
2000
1000
0
6
3
0
−2
−1
0
1
2

B

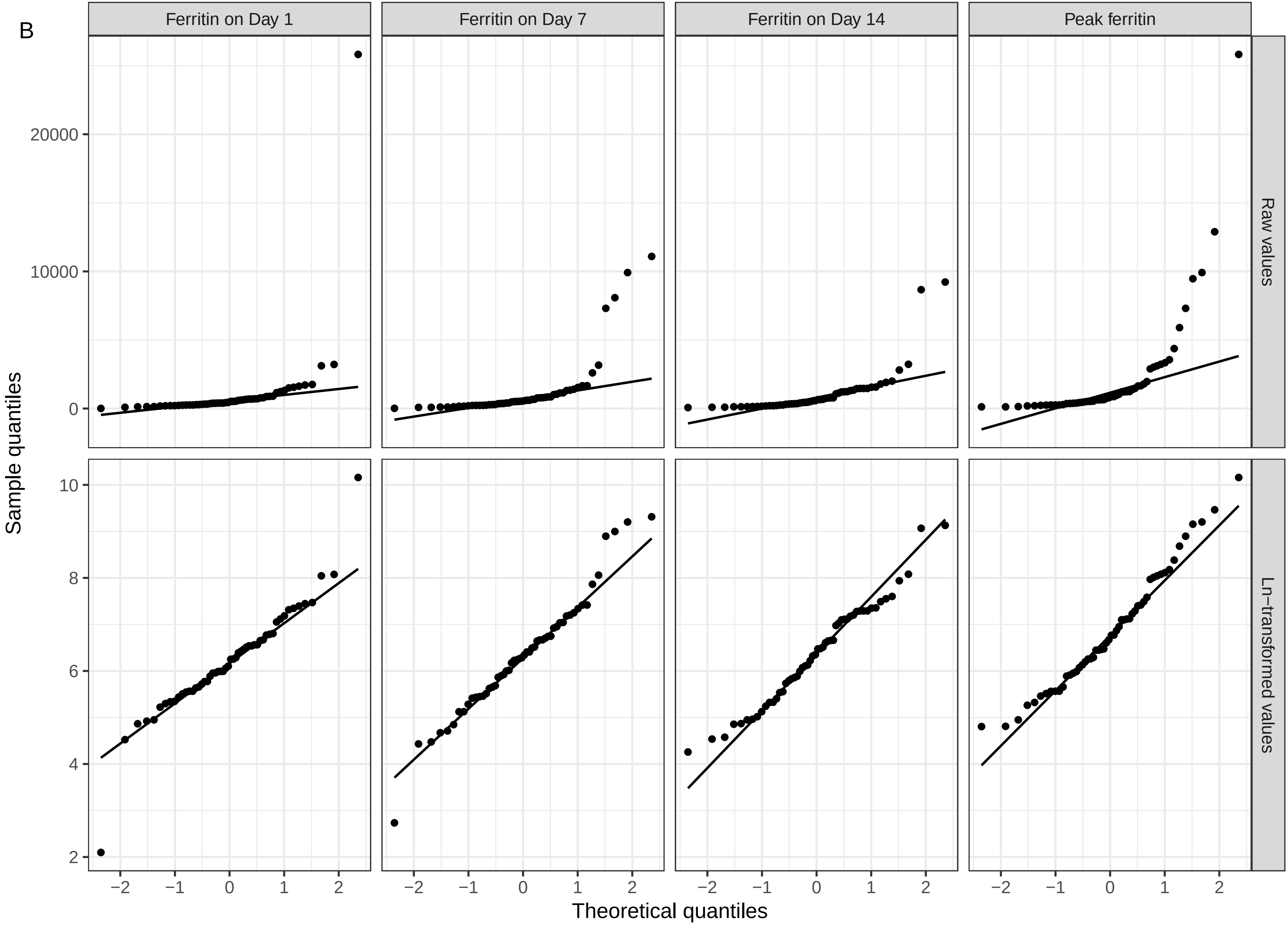
Ferritin on Day 1
Ferritin on Day 7
Ferritin on Day 14
Peak ferritin
Raw values
Ln−transformed values
Sample quantiles
Theoretical quantiles
20000
10000
0
10
8
6
4
2
−2
−1
0
1
2
